# Bradykinin‐mediated estrogen‐dependent depressor response by direct activation of female‐specific distribution of myelinated Ah‐type baroreceptor neurons in rats

**DOI:** 10.1111/cns.13792

**Published:** 2021-12-28

**Authors:** Ke‐Xin Li, Yan Feng, Xiong‐Xiong Fan, Xun Sun, Ying Li, Di Wu, Li Liu, Chang‐Peng Cui, Xue Xiong, Hu‐Die Li, Meng Zhou, Hai‐Lan Ma, Yang Liu, Rong Zhang, Bai‐Yan Li

**Affiliations:** ^1^ Department of Pharmacology (State‐Province Key Laboratories of Biomedicine‐Pharmaceutics of China, and Key Laboratory of Cardiovascular Medicine Research, Ministry of Education) College of Pharmacy Harbin Medical University Harbin China; ^2^ Department of clinical Laboratory The 1st Affiliated Hospital of Dalian Medical University Dalian China

**Keywords:** baroreceptor activation, bradykinin, depressor response, neurocontrol of blood pressure regulation, nodose

## Abstract

**Aim:**

To understand the direct impact of bradykinin in autonomic control of circulation through baroreflex afferent pathway.

**Methods:**

The mean arterial pressure (MAP) was monitored while bradykinin and its agonists were applied via nodose (NG) microinjection, the expression of bradykinin receptors (BRs) in the NG (1^st^‐order) and nucleus tractus solitarius (NTS, 2^nd^‐order) were tested in adult male, age‐matched female, and ovariectomized rats under physiological and hypertensive conditions. Additionally, bradykinin‐induced depolarization was also tested in identified baroreceptor and baroreceptive neurons using whole‐cell patch‐clamp technique.

**Results:**

Under physiological condition, bradykinin‐induced dose‐ and estrogen‐dependent reductions of MAP with lower estimated EC_50_ in females. B_2_R agonist mediated more dramatic MAP reduction with long‐lasting effect compared with B_1_R activation. These functional observations were consistent with the molecular and immunostaining evidences. However, under hypertensive condition, the MAP reduction was significantly less dramatic in N^’^‐Nitro‐L‐Arginine‐methyl ester (L‐NAME) induced secondary and spontaneous hypertension rats in males compared with female rats. Electrophysiological data showed that bradykinin‐elicited concentration‐dependent membrane depolarization with discharges during initial phase in identified myelinated Ah‐types baroreceptor neurons, not myelinated A‐types; while, higher concentration of bradykinin was required for depolarization of unmyelinated C‐types without initial discharges.

**Conclusion:**

These datasets have demonstrated for the first time that bradykinin mediates direct activation of baroreflex afferent function to trigger estrogen‐dependent depressor response, which is due mainly to the direct activation/neuroexcitation of female‐specific myelinated Ah‐type baroreceptor neurons leading to a sexual dimorphism in parasympathetic domination of blood pressure regulation via activation of B_2_R/B_1_R expression in baroreflex afferent pathway.

## Graphical abstract

Female‐specific subpolulation of myelinated Ah‐type baroreceptor neurons plays a critical role in BK/BKRs mediated autonomic control of BP regulation.

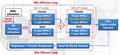

## 1 INTRODUCTION

The autonomic nerve system (ANS) is the key player in the blood pressure (BP) homeostasis via the balance between sympathetic (cardiac afferents) and parasympathetic (vagal afferents), in which vagal inputs dominate this balance by rendering negatively feedback reflexed control mechanism, the baroreflex (BRx) plays a major role in the neurocontrol of both short‐term and long‐term BP regulation. BRx afferent loop initiates the sensing BP fluctuation by baroreceptor terminals distributed in the aorta^1^ operated by mechanosensitive PIEZOs channels^2^, ^3^, ^4^ to the cell body of the 1^st^‐order neurons^5^, ^6^ in the nodose ganglia (NG), and then to the cell body of the 2^nd^‐order neurons^7^, ^8^ in the nucleus tractus solitarius (NTS) by forming the synapses through its central projection and the efferent loop innervates to the heart and blood vessel. Extensive animal studies have demonstrated that BRx afferent function is critical to stabilize the BP under physiological and hypertensive conditions,^7^, ^9^, ^10^, ^11^, ^12^, ^13^, ^14^ suggesting that dysfunction of BRx afferent function is crucial for the development of both primary and secondary hypertension.

The sex difference in BP has long been recognized between premenopausal women and aged‐matched men. The pathophysiological mechanism has been extensively explored that female hormones is one of the major mechanisms contributing to reduce the risk of cardiovascular complications.^15^ Moreover, a low‐threshold and sex‐specific distribution of myelinated Ah‐type baroreceptor neurons (BRNs) housed in NG and NTS has been identified,^16^ the neuroexcitability of this subpopulation depends upon the presence of estrogen (17β‐E2)^17^, ^18^, ^19^ and is regulated by neurotransmitter,^20^ which may impact on the sexual dimorphism of baroreflex afferent function and neurocontrol of circulation.^11^

Bradykinin (BK), a well‐known inflammatory mediator^21^ released from tissue damages, plays a role in renal protection^22^ and cardiovascular function as a key substance in the kallikrein‐kinin system (KKS),^23^ such as vasodilation and hypotension applied locally^24^ or systemically.^25^ This depressor response is supported by centrally application of BK directly into the nucleus ambiguous and NTS leading to the bradycardia^26^ and hypotension,^27^ while the pressor responses is produced by microinjection of BK directly into the paratrigeminal nucleus.^28^ Based upon the molecular weight of BK (1.06 KDa), however, it is hard to believe that BK itself can easily pass through the blood‐brain barrier (BBB)^29^ and modulate cardiovascular/vagal responses centrally, even though it can significantly increase the permeability of the BBB by activating B_2_R located on endothelial cells of capillary.^30^ So the questions remain that (1) what is the clinical impacts of this depressor or pressor response by centrally administration of BK; (2) whether the depressor response mediated by BK systemically or locally is a direct cause of baroreceptor activation initiated from BRx terminals of aorta; (3) how crucial of BK receptor expression in the NG and NTS to modulate the BRx under physiological and hypertensive disease condition; and (4) how important of female‐specific subpopulation of myelinated Ah‐type BRNs^11^, ^17^, ^31^, ^32^, ^33^ in BK‐mediated BRx afferent function. To answer these particular questions, the multiple in vivo and in vitro studies conjugated with electrophysiological and pharmacological approaches were used and the present data have demonstrated for the first time that microinjection of BK directly into the NG, housing the 1^st^‐order BRNs, produces marked depressor responses in females *vs*. age‐matched male and ovariectomized (OVX) female rats mainly through B_2_R under both physiological and hypertensive condition. Additionally, BK mediated the excitation of Ah‐type BRNs and higher expression pattern of B_2_R would be an afferent explanation of sexual dimorphism in BK‐mediated autonomic control of BP regulation.

## 2 MATERIALS AND METHODS

### 2.1 Animals

Age‐matched adult male and female Sprague Dawley (SD) rats 12–14 weeks weighing 220–250 g were purchased and licensed under SCXK (Hei) 2019–001 from the experimental animal center of the Second Affiliated Hospital of Harbin Medical University. Wistar‐Kyoto (WKY) and spontaneously hypertensive rats (SHR) (10 weeks weighing 180–200 g) were directly purchased from Vital River Laboratory Animal Technology Co., Ltd. with SPF grade and licensed under SCXK (2016–0006). All animals were maintained in a 12/12 h light cycle at 25°C with feeding food and water ad libitum. All animal protocols were pre‐approved by the Institutional Animal Care and Use Committee of Harbin Medical University, Harbin, China, which are in accordance with the recommendations of the Panel on Euthanasia of the American Veterinary Medical Association and the National Institutes of Health publication “Guide for the Care and Use of Laboratory Animals (http://www.nap.edu/readingroom/books/labrats/ ).

### 2.2 Chemicals

The chemicals involved in this experiment are listed in Table S1.

### 2.3 Hypertension models

SD rats were randomly divided into two groups. The hypertension group was induced by N'‐Nitro‐L‐Arginine‐methyl ester (L‐NAME),^9^ which was injected intraperitoneally (40 mg/kg/d) for 4 weeks.^34^, ^35^, ^36^ The systolic blood pressure (SBP) of all rats was measured weekly. The blood pressure above 135 mmHg is considered hypertension. The control group was administered intraperitoneally 2 ml/d of sterile saline solution.

### 2.4 Blood pressure measurement

The baseline blood pressures of all rats were measured for 2 weeks to accommodate the experimental environment and equipment interference. The SBP of all rats were measured weekly using a noninvasive blood pressure monitor (BP‐98A, Saffron,). The rats were placed in thermostatic (37°C) plastic holder. After adapting to experimental environment, the stable blood pressure about 5 values of each rat was recorded with tail‐cuff method^32^ and averaged values.

### 2.5 Surgical ovariectomy

The surgery was performed as earlier protocols.^37^ Anesthetized (3% pentobarbital sodium, 45 mg/kg^−1^) rats were placed in a lateral position and shaved both sides of their back. After disinfecting skin with 75% ethanol, a 2.0 cm incision was made from the 2^nd^ to 5^th^ lumbar vertebra on the left lateral side with a scalpel. The pink ovarian tissue was visible by lifting and pulling away gently the white adipose tissue. Ovary was removed after fixing between fallopian tube and ovary with hemostat forceps. Then, muscle layers and skin were sutured successively using 4–0 absorbable sutures and disinfected with iodophor. The other ovary was removed by the same procedure. Penicillin (80000 Units) was performed in each rat via intramuscular injection. After recovering from anesthesia, the rats were observed for at least 30 min to ensure that the surgical wounds were completely sutured before returning to the animal facility. Ovariectomized rats were housed for four weeks for subsequent experiments.

### 2.6 Baroreceptor sensitivity measurement

Following the previous protocol,^38^, ^39^ the rats were anesthetized with pentobarbital sodium. One cannula filled with heparin was inserted into the femoral artery and a pressure transducer (AD Instruments MLT 844, Norway) used to measure arterial pressure (MAP). Meanwhile, another cannula was inserted into femoral vein for drug administration. The electrocardiogram was monitored (LabChart 7 Pro software, AD Instruments, Bella Vista,). The sodium nitroprusside (SNP) and phenylephrine (PE) were injected intravenously at an incremental dose (1, 3, and 10 μg/kg), respectively. BRS (ΔHR/ΔMAP) was calculated by the maximum changes in HR and the associated MAP.

### 2.7 Nodose ganglion microinjection

This experiment was conducted by following the procedures as described previously.^14^, ^32^ Briefly, rats were anesthetized with chloralhydrate (10%, 0.4 g/kg, i.p.). Then, the femoral artery was cannulated to monitor the MAP. A 4.0 cm longitudinal midline incision was opened above the trachea. Under a stereo‐microscope (Olympus), the left side nodose ganglion (NG) was exposed. After the MAP tended to stabilize, 3 µl of bradykinin (BK), Sar‐[D‐Phe8]‐des‐Arg9‐bradykinin (B_1_R agonist), and [Phe8Ψ(CH‐NH)‐Arg9]‐bradykinin (B_2_R agonist) were injected into nodose ganglion using a precise microsyringe (Hamilton, O.D. × I.D. = 0.31 × 0.16), respectively. And then, the change of MAP was recorded.

### 2.8 Tissue preparation of nodose ganglion and nucleus tractus solitary

The NG was isolated as previously described by our laboratory method.^32^ Briefly, after losing the reflex response to tail pinch with 3% pentobarbital sodium intraperitoneal administration, the rats were quickly sectioned at the mid‐auxiliary region. Then, the entire nodose ganglion was acquired and placed into a Petri dish containing chilled (4°C) normal saline. Under a stereo‐microscope (Olympus), the surrounding connective tissue was gently removed and transferred to liquid nitrogen in order to molecular experiments.

The hindbrain was separated and placed in cold artificial cerebrospinal fluid 1 min. The bilateral medulla was trimmed to a 1‐cm block (rostral‐caudal) centered on the obex under a microscope.^40^ The region containing nucleus tractus solitary (NTS) was stored at −80°C for further molecular investigation.

### 2.9 Quantitative real‐time PCR

The mRNA expression of B_1_R and B_2_R was determined using qRT‐PCR. Following the manufacturer's instructions, the total RNA was extracted using the TRIzol^®^ Reagent. And then cDNA was synthesized with ReverTra Ace qPCR RT Kit using RNA as a template. The SYBR Green PCR Master Mix Kit was used to quantify target genes using an ABI QuantStudio 6 Flex real‐time PCR system (Applied Biosystems by Thermo Fisher Scientific,). GAPDH was used as an internal control. Data were analyzed with 2^−ΔΔCt^ method.^41^ The primers (Invitrogen, Frederick,) used in this experiment are listed in Table S2.

### 2.10 Western blot analysis

According to the manufacturer's instructions, the total protein was extracted in protein lysate buffer (RIPA: SDS: PI, 60: 40: 1) from isolated NG and NTS. Then supernatant from the centrifugal lysate was measured by a BCA Protein Assay Kit. The protein samples (NTS: 100 μg, NG: 120 μg) which were boiled for 5 min were separated on 10% SDS‐PAGE and were transferred from the gel onto nitrocellulose membranes. After completing that, the membranes were immersed in a blocking solution of 5% (g/ml) skim milk powder for 2 h, and then the membranes were incubated overnight at 4°C with primary antibodies of B1R, B2R, and GAPDH. The appropriate secondary antibodies (1:8000, antirabbit/antimouse) were incubated for 55 min at room temperature. The protein blots on membranes were finally detected using Odyssey Infrared Imaging System (#ODY‐3149, LI‐COR, Lincoln). The antibodies used in this experiment are listed in Table S3.

### 2.11 Immunohistochemical analysis

As described in our previous experiments,^32^, ^42^ fixed tissues with 4% buffered paraformaldehyde were placed in 30% sucrose at 4°C overnight. Then, tissues were put in embedding medium and frozen at −80°C for 1 h before being sectioned into a thickness of 10 µm using the cryostat (LEICA cm 1850). The histological sections were covered penetrating solution (10% BSA and 3% Triton‐X in PBS) and blocked with Goat Serum for 2 h at 37°C. Then tissues were incubated with the primary antibodies of B1R or B2R in PBS containing 20% BSA, 10% Goat Serum, and 6‰ antibody of HCN1 at 4°C overnight. After that, the appropriate secondary antibodies (1:200, Alexa Fluor^®^ 488 goat antirabbit/594 goat antimouse) cocktail consisting of 20% BSA and 10% goat serum in PBS were used to incubate for 1 h at 37°C. The nuclei were stained with DAPI (1: 30) for 30 min at room temperature. After covering the slides, imaging was visible under confocal microscope (#37081, Carl Zeiss,). The involved antibodies are listed in Table S3.

### 2.12 Electrophysiology

For action potential (AP) recordings, the composition of the intracellular solution was (in mM): NaCl 10; KCl 50; K_2_SO_4_ 50; MgCl_2_ 5.0; 10.0 HEPES, pH adjusted to 7.25 using 1 N KOH. Immediately prior to filling the patch pipettes, 2.0 Mg‐ATP and 2.0 Na‐GTP were added to the pipette solution (in mM) along with 4.0 BAPTA‐Na and 0.25 CaCl_2_ for a final buffered [Ca^2^ ^+^]_i_ of 100 nM. The composition of the extracellular recording solution was (in mM): NaCl 137; KCl 5.4; MgCl_2_ 1.0; CaCl_2_ 2.0; glucose 10; HEPES 10, pH adjusted to 7.30–7.35 using 1 N NaOH. The osmolarity of the extracellular and intracellular solutions was adjusted to 310–315 and 290–295 mmol/kg, respectively, using D‐manitol. Whole‐cell patch recordings were performed using the Axopatch 200B or MultiClamp 700A amplifier (Axon Instruments,). Borosilicate glass pipettes (Sutter Instruments,) were pulled and polished down to 1.5–2.4 MΩ. Following the formation of a GΩ seal, the pipette capacitance was compensated. The total cell capacitance (30–40 pF) and electrode access resistance (3–5 MΩ) were also compensated 60%–80%. All patch experiments were conducted at room temperatures (22–23°C). Data traces were low pass filtered to 10 kHz and digitized at 50 kHz using pCLAMP 9.0/10.2 (Axon Instruments,) and Digidata 1322A/1440A (Molecular Devices,) operating on a PC platform.

Two distinct experimental protocols were performed in current‐clamp mode. Firstly, a single AP was elicited by applying a brief (<500 ms) super threshold current pulse through the patch electrode. The purpose of protocol is to identify afferent fiber type of isolated neurons into three classifications, ie, myelinated A‐, Ah‐, and unmyelinated C‐types^5^, ^6^, ^31^ based upon the waveform characteristics, such as the AP firing threshold (APFT), the AP upstroke velocity measured at 50% peak‐to‐peak excursion (UVAPD_50_) and the AP downstroke velocity (DVAPD_50_). Secondly, a gap‐free protocol was utilized to test the membrane depolarization from the resting membrane potential (RMP) in the presence of BK. Before recording, the RMP was adjusted to ~60 mV by current injection to scandalized condition. The time of duration was determined by the maximal depolarization.

### 2.13 Data analysis

Clampfit (Molecular Devices;) was used for initial data readings and excel for statistical analysis (Microsoft,). Trace filtering and data graphing were accomplished by origin (Microsoft,). The test for normal distribution of the collected results was performed routinely by using the origin software with the formality test (Shapiro‐Wilk), and the results showing normal distribution were further analyzed using either *t*‐test or ANOVA. Both unpaired and paired *t*‐test was used to compare the difference between groups or the difference before and after treatment; one‐way ANOVA with post hoc Turkey test was also selected where appropriate to compare the difference among groups. The averaged data were presented as mean ± SD unless specified elsewhere. The *p* value of equal or less than 0.05 was considered statistically different.

## 3 RESULTS

### 3.1 Sexual dimorphic and estrogen‐dependent BP reduction induced by BK microinjection directly into the NG

**FIGURE 1:**
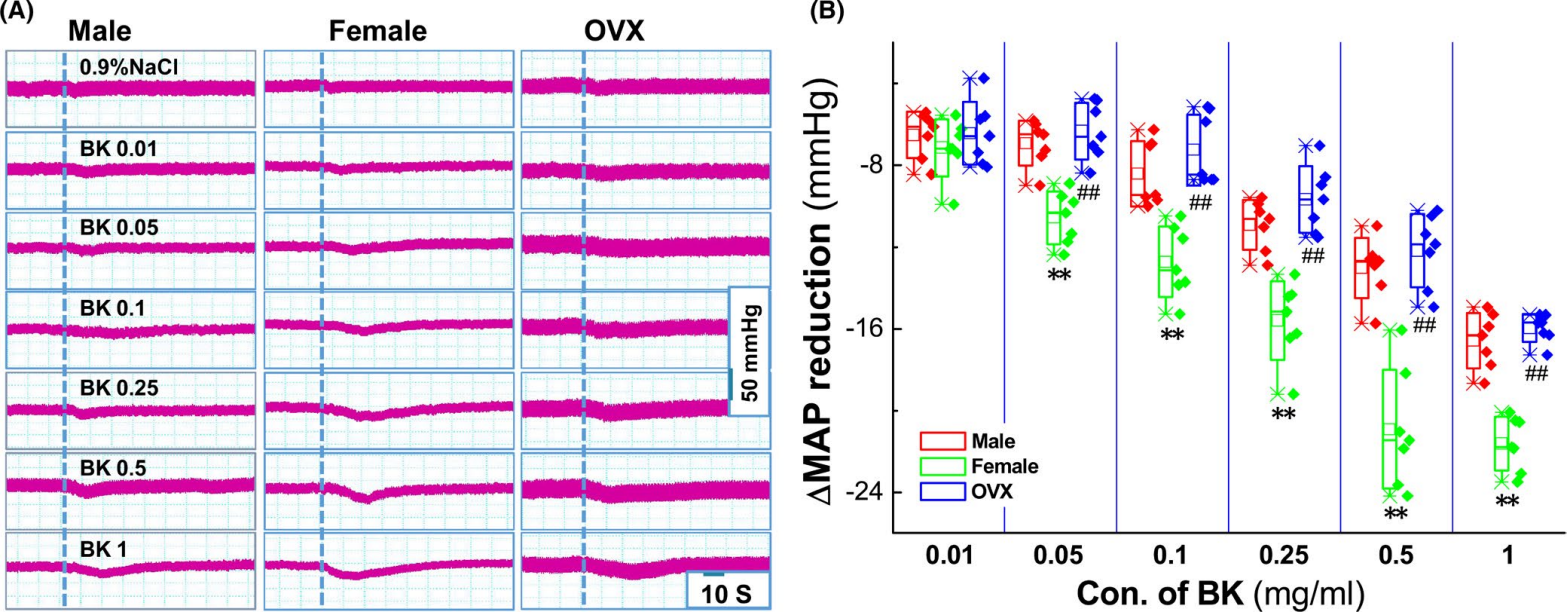
Sexual difference in Bradykinin (BK)‐mediated mean arterial pressure (MAP) reduction by nodose ganglion (NG) microinjection under physiological condition. The femoral artery cannulation was performed, and MAP was collected before and after administration with 0.9% NaCl (vehicle control) and different concentrations of BK (mg/ml), respectively. (A) The representative recordings of BK‐mediated MAP reduction in the presence of a series of dosage of BK in adult male (left), age‐matched female (middle) and ovariectomized (OVX, right) rats. Dot lines represent the time of the beginning of microinjection. The scale bars applied for all recordings; (B) Summarized data of the changes in MAP. Averaged data were presented as mean ± SD, **p < 0.01 *vs*. Male, ^##^ *p* < 0.01 *vs*. Female, *n* = 7 rats/group

**FIGURE 2:**
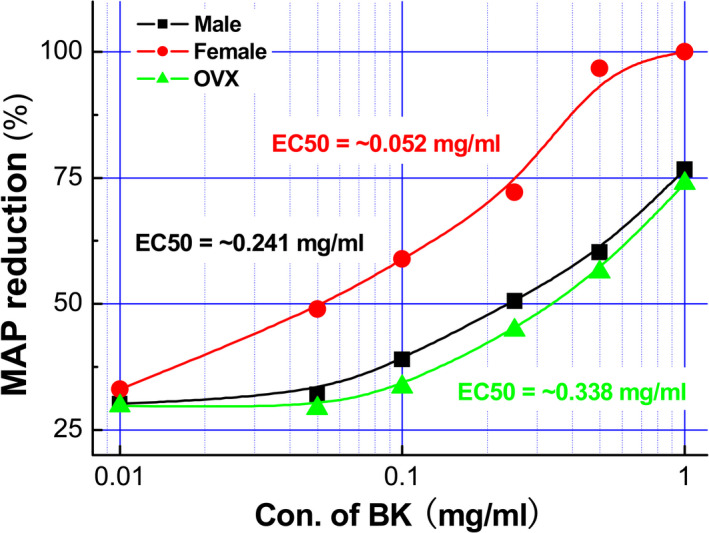
The dose‐response curves and EC_50_ of BK‐mediated MAP reduction when microinjection of BK directly into the NG. The changes in mean arterial pressure (MAP) were recorded while bradykinin (BK) was microinjected directly into the nodose (NG) with different dosages of BK (0.01, 0.05, 0.1, 0.25, 0.5, and 1 mg/ml, 3 μl). Taking the BK concentration as the abscissa and the percentage of BP reduction as the ordinate, the dose‐response curves of BK were established in male (black), female (red), and OVX (green) rats, *n* = 7 rats/group. Estimated EC_50_ was ~0.241, ~0.338, and 0.052 mg/mL, respectively, for male, female, and OVX group

BK plays an essential role in the vasodilation and development of the BP phenotype. To assess the role of the baroreflex afferent function, a series of concentration of BK (0.01~1.0 mg/ml) was applied to the NG directly through microinjection, and the results showed that, compared with the control (0.9% saline), BK caused significant BP reduction (Figure S1 and Table S4) in a concentration‐dependent manner in adult male, age‐matched female, and ovariectomized (OVX) female rats (Figure 1A). Interestingly, the reduction in the MAP was more dramatic in the females than that observed in age‐matched male rats (Figure 1B), and this sexual difference in BP reduction was disappeared at all concentrations of BK when bilateral ovaries were removed surgically, indicating the involvement of estrogen in BK‐mediated BP reduction under physiological condition. Additionally, by looking into the value of 50% of the efficacy (estimated EC_50_) of BK, it was almost 5 to 6 folds higher in the male (~0.241 mg/ml) and OVX (~0.338 mg/ml) rats compared with ovary intact females (~0.052 mg/ml) (Figure 2), suggesting that less concentration of BK is required to reach a similar response (BP reduction). Figures 1, 2, Figure S1, and Table S4

### 3.2 Dominative role of the type‐II BK receptor in BK‐mediated BP reduction

**FIGURE 3:**
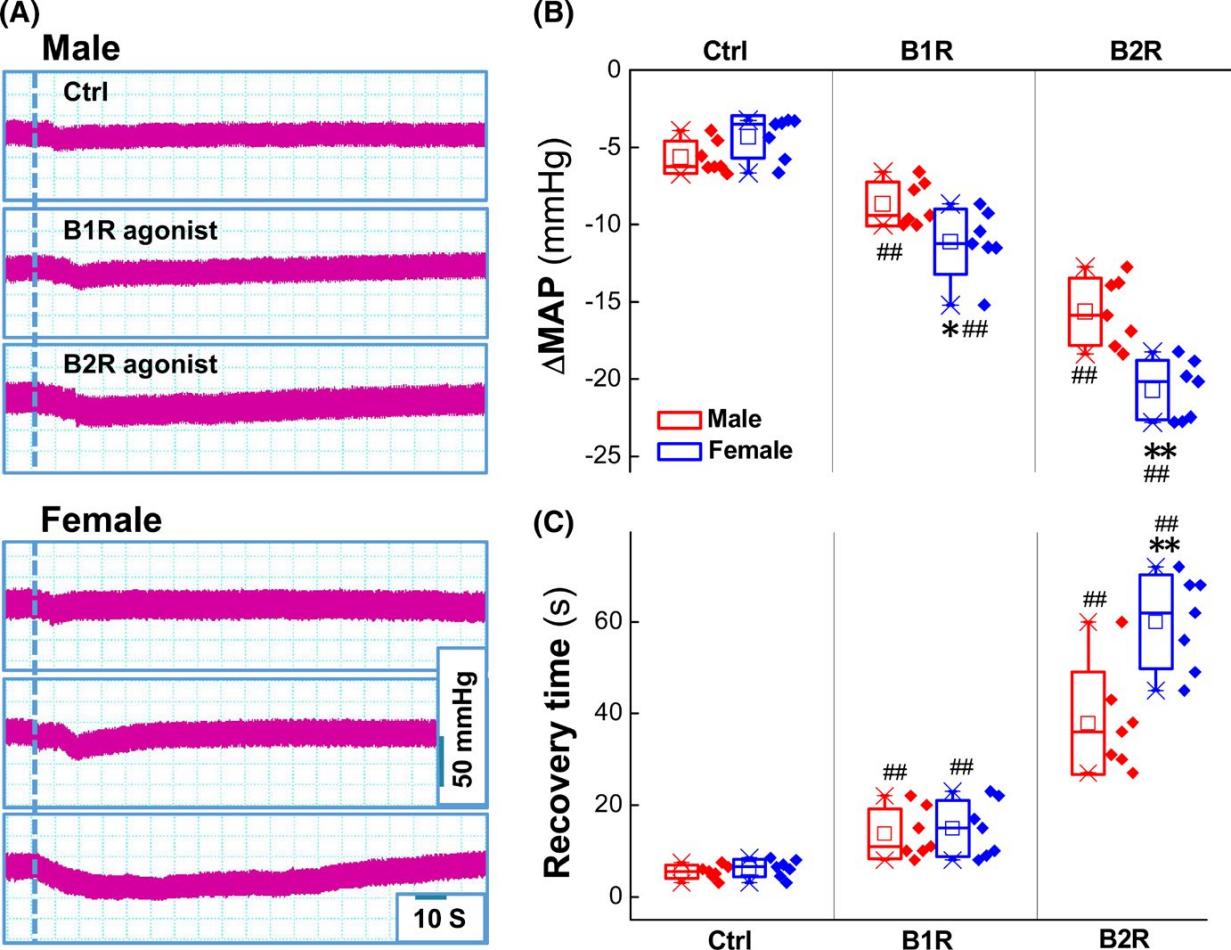
The effect of bradykinin receptor agonists (BRs) on mean arterial pressure (MAP) reduction. (A) The representative recordings of the MAP in the presence of 3 μg B1R selective agonist (Sar‐[D‐Phe^8^]‐des‐Arg^9^‐Bradykinin) or 3 μg B2R selective agonist ([Phe^8^Ψ(CH‐NH)‐Arg^9^]‐Bradykinin); the dash line means the time of application; the scale bars applied for recordings; (B‐C) Summarized data of the changes in MAP (mmHg) and the recovery time (sec) in the presence of B1R or B2R agonist. Averaged data were presented as mean ± SD, *p < 0.05 and **p < 0.01 *vs*. male, ^##^ *p* < 0.01 *vs*. Ctrl, *n* = 7 rats

To test the role of BK receptor (B_1_R or B_2_R) activation in BK‐mediated BP regulation, the selective agonists for both B_1_R (Sar‐[D‐Phe8]‐des‐Arg9‐bradykinin) and B_2_R ([Phe8Ψ (CH‐NH)‐Arg9]‐BK) were microinjected (1 mg/ml, 3 μl) directly into the NG under the similar condition. Notably, B_2_R agonist induced more dramatic and long‐lasting BP reduction (Table S5), while B_1_R agonist caused a brief and transient BP reduction. Additionally, these effects mentioned above were more potent in females than those in age‐matched male rats (Figure 3A–C), suggesting that B_2_R is the key player in BK‐mediated and sexual‐dimorphic BP reduction via baroreflex afferent function. Figure 3 and Table S5

### 3.3 Estrogen‐dependent expression of BK receptors in the NG and NTS

**FIGURE 4:**
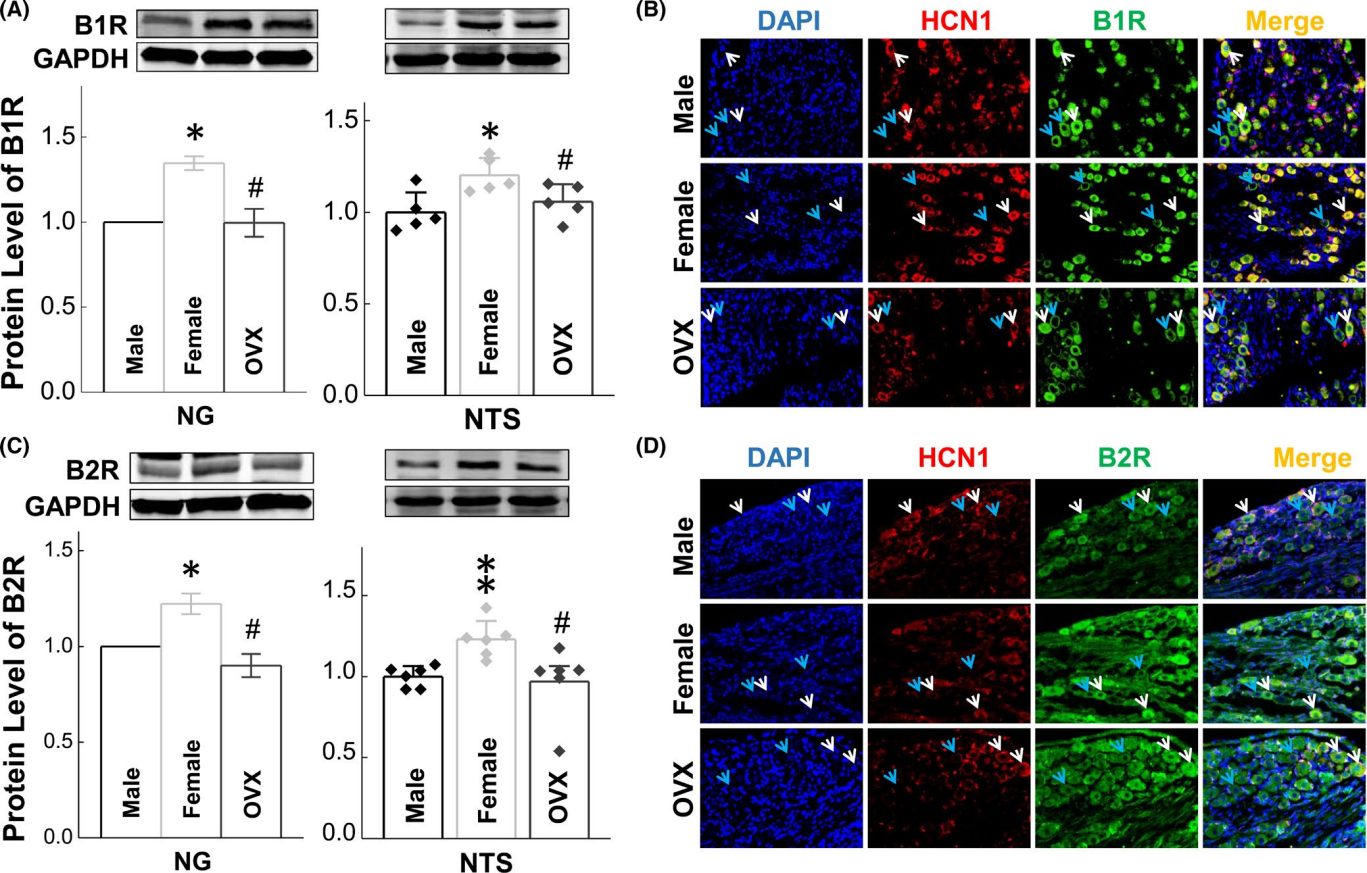
Bradykinin receptors (BRs) expression in the nodose ganglia (NG) and nucleus tractus solitary (NTS) in male, age‐matched female, and ovariectomized (OVX) rats. The protein expression and distribution were assessed by western blot and immunostaining with specific antibody against B1R or B2R. White and orange arrowheads: HCN1‐positive (myelinated A‐ and Ah‐types) and HCN1‐negative (unmyelinated C‐types) neurons in the NG; (A‐B) the protein expression and distribution of B1R in the NG and NTS tissues; (C‐D) the protein expression and distribution of B2R in the NG and NTS tissues; Averaged data were presented as mean ± SD; *p < 0.05 and **p < 0.01 *vs*. male; ^#^ *p* < 0.05 and ^##^ *p* < 0.01 *vs*. female (mRNA: *n* = 4–5 from 4–5 rats, protein: *n* = 3–6 from 6–12 rats)

Functional study has demonstrated that BK mediates stronger response in female rats by direct baroreflex activation, suggesting more functional expression of B_1_R and/or B_2_R in the baroreflex afferent pathway of female rats. As expected, the relatively higher expression of both B_1_R (Figure 4A) and B_2_R (Figure 4C) were confirmed in the NG and NTS of female compared with age‐matched male rats, while the protein levels of both B_1_R and B_2_R were downregulated in the OVX female rats *vs*. ovary intact females to the identical levels of male rats. Additionally, similar trend of mRNA expression for either B_1_R or B_2_R was also observed in the NG and NTS (Figure S2). The functional distribution of B_1_R and B_2_R was also confirmed by immunostaining analysis with the antibodies against B_1_R and B_2_R (Figure 4B,4D). Additionally, the result of co‐localization of HCN1 and BK receptors indicated that both myelinated (white arrowheads: HCN1‐positive and presumably A‐ and Ah‐type) and unmyelinated (orange arrowheads: HCN1‐negative and presumably C‐type) neurons were all expressed B_1_R and B_2_R in male, age‐matched female, and OVX rats. Even though we could not distinguish A‐ from Ah‐types under current experimental condition, the percentage of myelinated neurons (A‐ plus Ah‐type: over 50% of total pointed) in adult females was significantly higher than the normal distribution (10%–15% of total for A‐ and Ah‐type, respectively and 70% for C‐types), suggesting that myelinated HCN1‐positive neurons definitely contain both A‐ and Ah‐types; Most importantly, with higher magnification of immunostaining images (Figure 4B,4D), the fluorescent intensity for both B_1_R and B_2_R of myelinated HCN1‐positive neurons (A‐ and Ah‐type) were significantly higher compared with age‐matched male and OVX female rats, strongly indicating that a female‐specific subpopulation of myelinated Ah‐type BRNs is very likely to express relatively higher level of BK receptors and that would be a potential reason to lead the sexual dimorphism in autonomic control of BP regulation. To further confirm the involvement of myelinated Ah‐type in BK‐induced BP reduction, the membrane depolarization was tested using whole‐cell patch technique in the presence of BK. Figure 4, Figure S2

### 3.4 Hypotensive action by direct activation of BK receptors in L‐NAME and spontaneously hypertension rats

**FIGURE 5:**
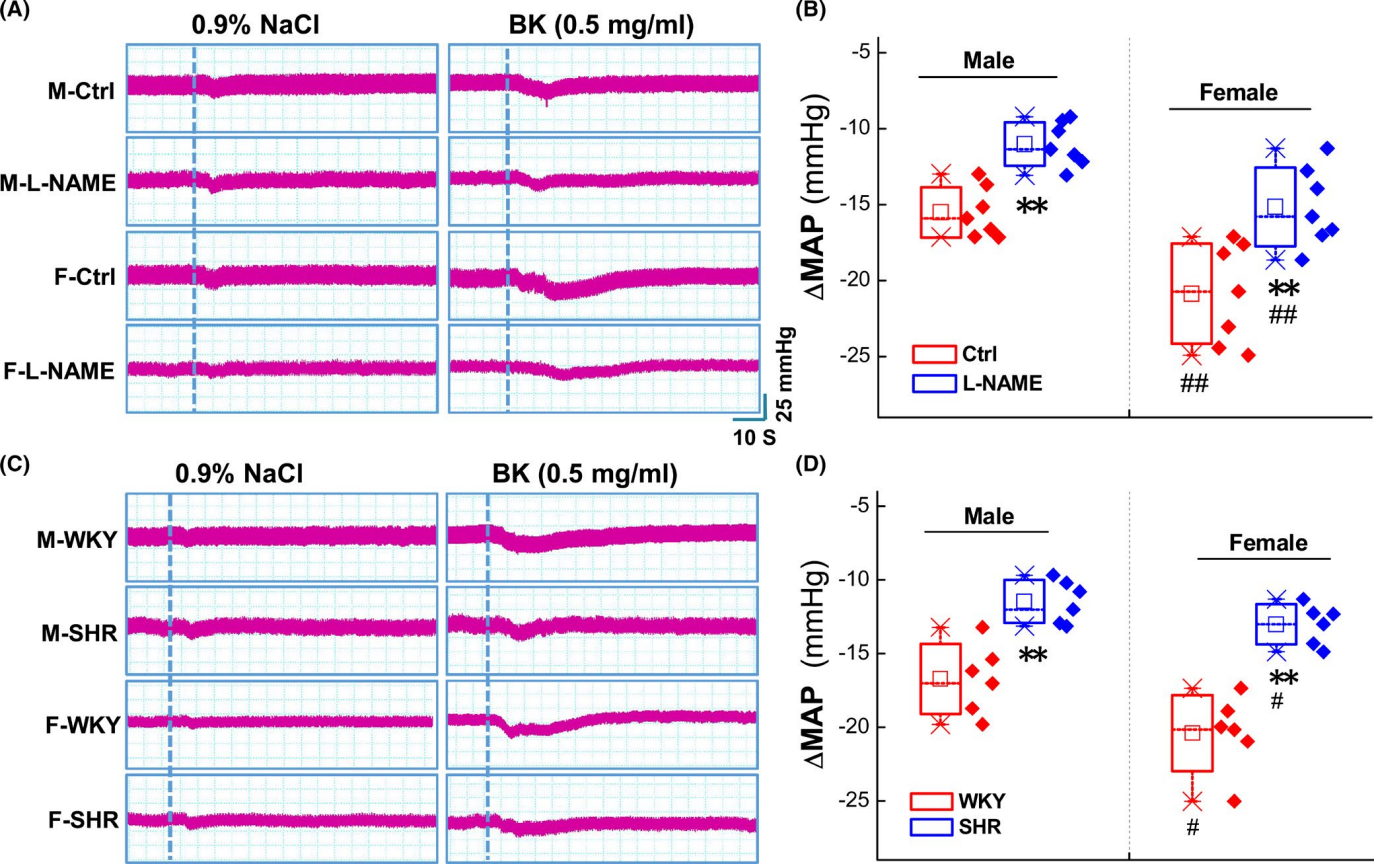
Changes in MAP after microinjection BK into the NG of secondary (L‐NAME) and primary (spontaneously) hypertension model rats. (A) The representative MAP recordings before and after administration with 0.9% NaCl and BK (0.5 mg/ml) in L‐NAME model rats. The dotted line indicates the time of the beginning of treatment. (B) Summarized data for the ΔMAP values after BK microinjection. Averaged data were presented as mean ± SD; **p < 0.01 *vs*. Ctrl, ^##^ *p* < 0.01 *vs*. Male, *n* = 7 rats. (C) The MAP recordings before and after administration with 0.9% NaCl and BK (0.5 mg/ml) in male and female WKY and SHR rats. The representative MAP recordings after microinjection and the dotted line indicates the time of the beginning of application. (D) Summary data for the ΔMAP values before and after BK microinjection. Data were represented as mean ± SD. **p < 0.05 *vs*. WKY, ^#^ *p* < 0.05 *vs*. Male, *n* = 6

The hypotensive action of BK has been confirmed via direct activation of BRs under physiological condition in either male or female rats. Whether or not this action could be observed under hypertensive condition of both primary and secondary hypertension needs to be answered. To this end, L‐NAME (Figure S3A) and spontaneously hypertension rats (SHR, Figure S3B) with either sex were selected and the results showed that, by microinjection of 0.5 mg/ml BK into the NG, the MAP was obviously reduced soon after BK application in both secondary (Figure 5A) and primary hypertension rats (Figure 5C). Notably, averaged data showed that although the hypotensive action of BK remained but significantly less in BK‐treated model rats compared with control group of male (Figure 5B) and female rats (Figure 5D), consistent with the impairment of baroreflex afferent function as revealed by the baroreceptor sensitivity (BRS) in both hypertensive models (Figures S4 and S5). Additionally, BP reduction before and after BK microinjection were more potent in female L‐NAME and SHR rats compared with age‐matched male rats, which is likely to be associated with the downregulation of BK receptors in baroreflex afferent pathway. Figure 5, Figures S3–S5.

### 3.5 Downregulated expression of BK receptors in the NG and NTS in L‐NAME hypertension rat models

**FIGURE 6:**
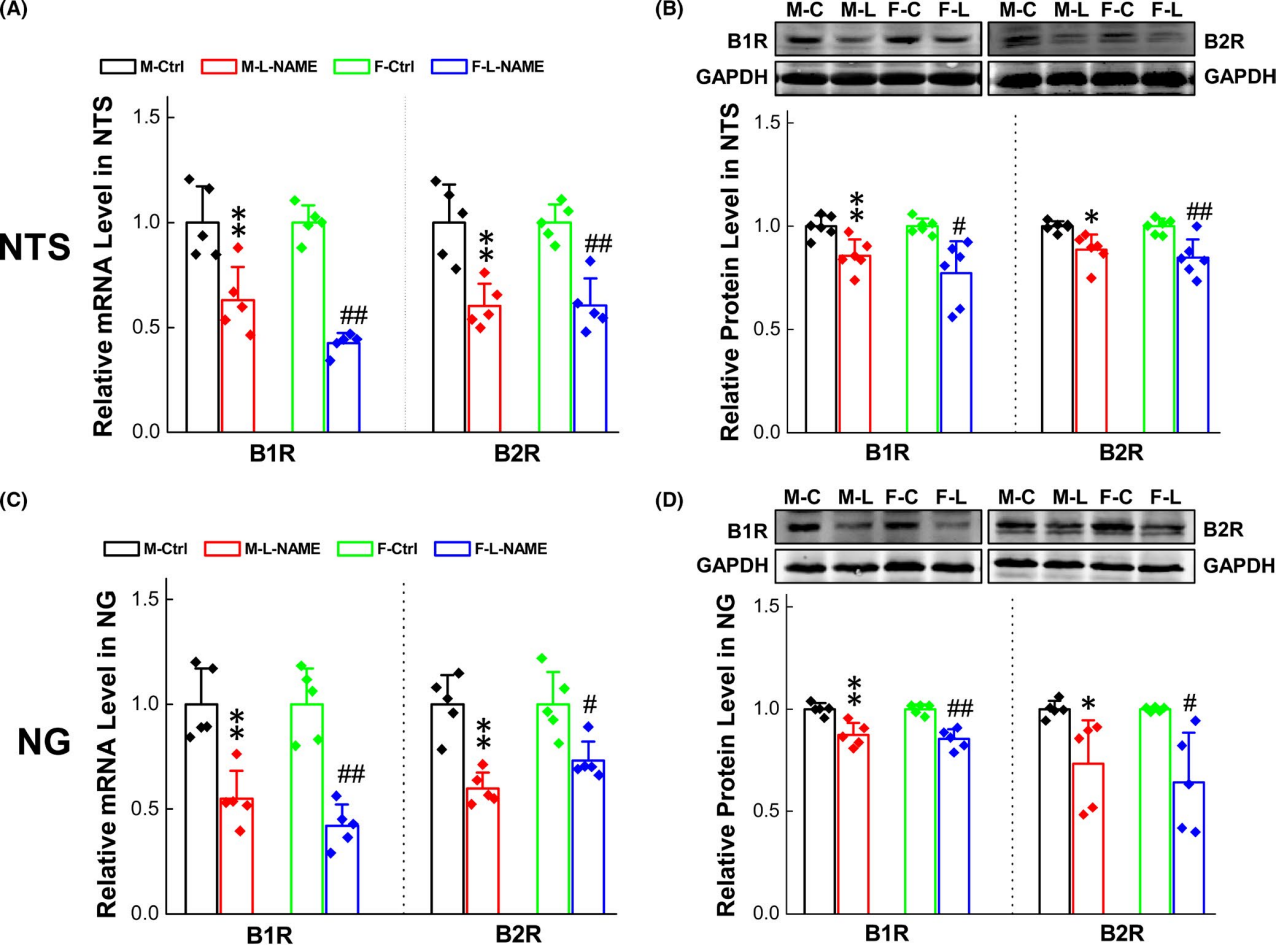
Changes of BRs expression in secondary (L‐NAME) hypertension model rats. (A‐B) B1R/B2R expression changes in mRNA and protein levels at the tissue of NTS and NG in the secondary hypertension model rats. Date were presented as mean ± SD; *p < 0.05 and **p < 0.01*vs*. M‐Ctrl, ^#^ *p* < 0.05 and ^##^ *p* < 0.01 *vs*. F‐Ctrl. (mRNA: *n* = 4 from 4 rats, protein: *n* = 5–6 from 10–12 rats)

The functional observation has pointed out that the BK‐induced hypotensive action is significantly reduced in the hypertensive disease condition compared with their control rats, so we have strong reason to believe that this is likely to be attributed to the downregulation of B_1_R/B_2_R expression in baroreflex afferent pathway. To test this, both qRT‐PCR and immunoblotting were employed, and the results was consistent with our hypothesis showing that both B_1_R and B_2_R were significantly downregulated, respectively, in either sex at the tissue level of NG (Figure 6C,D) and NTS (Figure 6A,B), suggesting that the downregulated B_1_R and B_2_R are the key factor to be attributed to the less hypotensive effects of BK in the hypertensive condition. Although, the sexual dimorphism in BK‐mediated BP reduction is closely associated with estrogen‐dependent expression of B_1_R and B_2_R; however, which type of baroreflex afferent neurons are the key in BK‐mediated effect needs to be answered at cellular level, such as single‐cell qRT‐PCT or patch‐clamp technique with identified neurons isolated from adult female rats. Figure 6

### 3.6 Afferent explanation of BK‐mediated neuroexcitation

Even though single‐cell qRT‐PCR has been conducted in our previous observation, the negative result and large variation among cells are often observed due largely to the amplification procedures of PCR; also the positive expression may not reflect real situation from a functional point of view. Thus, whole‐cell patch recording (membrane depolarization or inward current) from isolated NG neurons identified by electrophysiological and pharmacological validations in the presence of BK or its agonists would be perfect for afferent explanation of BK‐mediated BP regulation.

**FIGURE 7:**
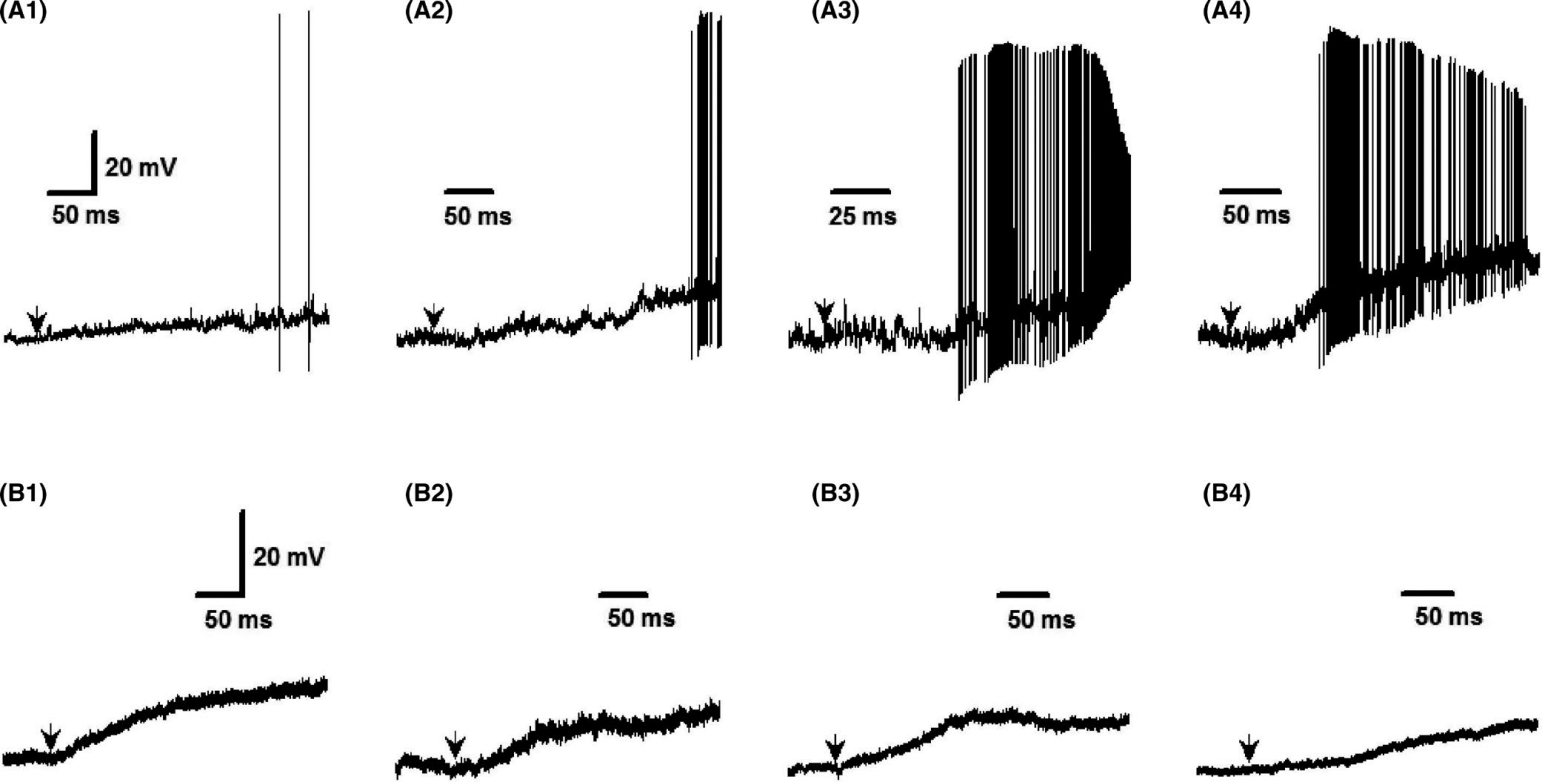
Bradykinin‐induced inward currents in the nodose neurons acutely isolated from adult female rats. The inward currents were recorded under voltage‐clamp configuration with gap‐free protocol in the neurons identified by electrophysiological validation, such as the action potential (AP) firing threshold, AP duration, the maximal up and downstroke velocity with or without the repolarization hump (the details see the Figure S7). (A1‐A4): The four representative recordings of inward currents with significant instant firing during the depolarization in the presence of 30 nM of BK (arrowheads) in identified myelinated Ah‐types; (B1‐B4): The four representative recordings of inward currents without significant instant firing during the depolarization in the presence of 300nM of BK (arrowheads) in identified unmyelinated C‐types. The vertical scale bar in the (A1) and (B1) apply for all other recordings

Based upon the functional and molecular observations, it is easy to believe that female‐specific subpopulation of myelinated Ah‐type BRSs would be a key player in BK‐mediated sexual dimorphism in hypotensive action (Figures 1,2). If this is the case, a higher sensitivity of Ah‐type BRNs to BK (less concentration of BK) to induce a membrane depolarization was highly expected. Additionally, the immunostaining observation could not distinguish the role of myelinated A‐ and Ah‐types in BK‐induced BP reduction even though the fluorescence was detected in HCN1‐positive neurons (Figure 4B,4D). To test this hypothesis and answer this particular question, whole‐cell patch experiments were conducted under whole‐cell configuration with both current‐ and voltage‐clamp modes, and single action potential (AP) was elicited by a brief pulse and the derivative current changes over the course of membrane potential was analyzed to verify the afferent fiber type (Figure S6) of neurons isolated from adult female rats with aortic depressor nerve labeled with Dil. Under this experimental condition, the membrane depolarization and inward currents were recorded in the presence of BK (10~300 nM) with bath perfusion in identified neurons (Figure 7).

**FIGURE 8:**
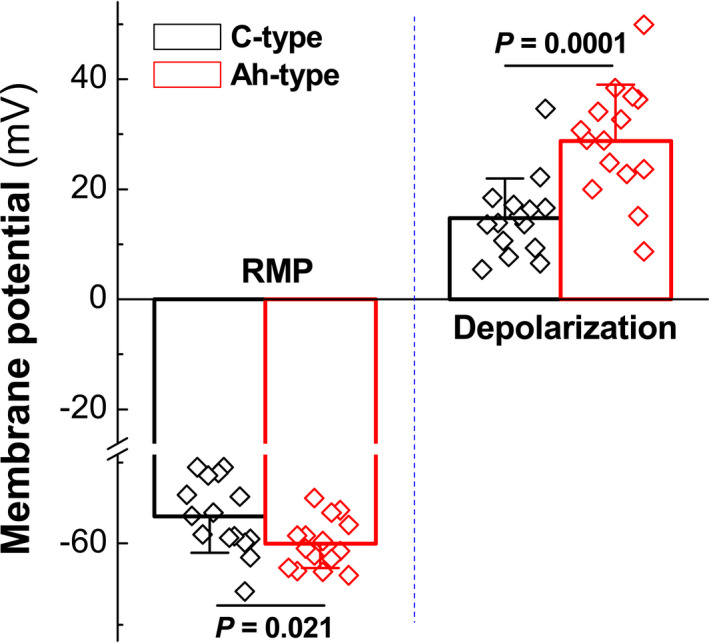
Summarized data for bradykinin (BK)‐induced membrane depolarization in both identified myelinated Ah‐ and unmyelinated C‐type nodose ganglia (NG) neurons isolated from adult female rats. The representative tracings are shown in Figure 7, and the inward currents were recorded in the presence of 30 nM and 300 nM of BK, respectively, in Ah‐ and C‐types. Averaged data were presented as mean ± SD, *n* = 15 recordings from at least 6 preparations

Interestingly, all tested A‐type BRNs were not responded to BK application under all BK concentrations (data not shown). While, based upon the pilot observation, 30 nM of BK was a minimal concentration of BK to induce the depolarization in all tested Ah‐type neurons (Figure 7A1–A4, four representatives) and repetitive firing could be detected in all identified Ah‐types. For C‐types, 300 nM of BK was necessary to evoke membrane depolarization in all tested C‐types (Figure Bb1–B4, four representatives) without repetitive firing during the course of depolarization. Importantly, Ah‐types not only required less concentration of BK for depolarizing the membrane potential but also showed the potent responses to BK (Figure 8, *p* < 0.0001, *n* = 15) as well compared with C‐types (*n* = 15). This observation highly suggests that myelinated Ah‐type BRNs are the key player in the sexual dimorphism in BP regulation via baroreflex afferent pathway due mainly to their sex‐specific distribution and sensitivity to BK, rather than A‐ and C‐type.

To further verify the role, BK‐induced inward currents were also observed and 30 nM of BK indeed induced a significant inward currents (Figure S7, *n* = 16) in myelinated Ah‐types, but not myelinated A‐types (data not shown), with dramatic repetitive Na^+^ channel activation during the initial phase of inward currents. Similarly, the 300 nM of BK‐induced inward currents were also observed in unmyelinated C‐types with significantly less response to BK without repetitive Na^+^ channel activation (Figure S8, *n* = 16). Figures 7 and 8, Figure S6–S8

## 4 DISCUSSION

**FIGURE 9:**
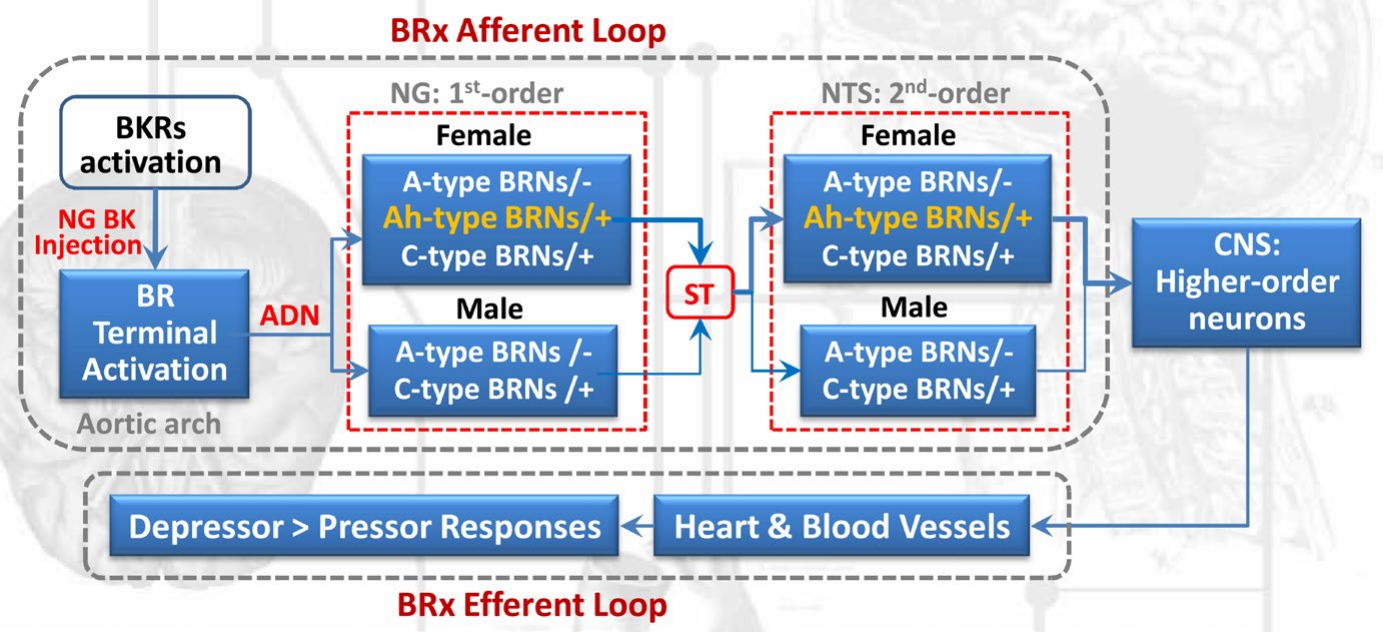
Schematic diagram of BK/BKRs‐mediated autonomic control of BP regulation (depressor responses) via baroreflex afferent activation. ADN: aortic depressor nerve; BK: bradykinin; BKRs: bradykinin receptors; BR: baroreceptor; BRx: baroreflex; BRNs: baroreceptor neurons; CNS: central nervous system; NG: nodose ganglion; NTS: nucleus tractus solitaries; ST: solitary track

Based upon the current observations (Figure 9), our major novel findings are (1) BRNs activation by microinjection of BK into the NG causes significant BP reduction in a concentration‐dependent manner, which is more dramatic in an intact females compared with age‐matched males and OVX female rats; (2) B_2_R activation is the key player in BK‐mediated BP reduction with estrogen‐dependent feature; (3) consistently, estrogen‐dependent expression of B_1_R and B_2_R are also confirmed in the tissue level of NG and NTS under physiological condition; (4) BK‐mediated BP reduction becomes less dramatic in males *vs*. age‐matched female rats in primary and secondary rat models of hypertension with consistent downregulation of B_1_R and B_2_R; (5) significantly lower concentration is required to induce the membrane depolarization with extensively repetitive firings during the initial phase in female‐specific distribution of myelinated Ah‐type BRNs (30 nM), rather than myelinated A‐types, compared with unmyelinated C‐types (300 nM). Figure 9

Even though central applications of BK causes depressor^26^, ^27^ or pressor^28^ responses, it may have no clinical significance simply because of its molecular weight of BK over 1.0 kDa that is hard to believe that it can easily passing through the BBB. Therefore, the hypotensive effect of BK due to the vasodilation by systemic administration would be the real pharmacological action of BK. However, direct action of BK on BRx, especially BRx afferent function, has not been well evaluated. Firstly, the plasma concentration of BK has been excluded for its differential role in vasodilation‐mediated BP reduction under physiological and hypertensive condition because there is no significant difference between normotensive subjects and patients with hypertension.^43^ This observation comes into a clue that BRx afferent function is likely the key player in BK‐mediated BP reduction with a concentration‐ and estrogen‐dependent fashion mainly through B_2_R activation due at least to the binding affinity. Intriguingly, this hypothesis has been confirmed by direct activation of BRNs with BK or its agonists. Notably, in order to understand the sexual dimorphism in BK‐induced BP reduction, whether there is a sex difference in a plasma concentration of BK needs to be answered and the study^44^ has demonstrated that the concentration of BK and BK‐1–5, as well as tissue plasminogen activator are similar among premenopausal, postmenopausal women, and men during BK infusion, suggesting that the sexual‐related expression pattern and modification of BK receptors would be more important and clinical significance in the BK‐mediated BP reduction via BRx afferent pathway under both physiological and hypertensive condition. Additionally, reduced BK‐mediated BP reduction in primary and secondary hypertensive model rats is also likely to be due at least in part to the modification of BK receptor, rather than the changes in plasma BK concentration. It is noteworthy that L‐NAME, as a non‐specific nitric oxide synthase inhibitor, has been widely reported to induce hypertension. Nitric oxide is one of the well‐recognized agents, which interacts with morphine in the central nervous system (CNS). It not only plays the role in morphine analgesia, tolerance, and dependence^45^ but also is involved in a spectrum of fundamental intracellular events that lead to vasorelaxation, inhibition of platelet aggregation, endothelial regeneration, suppression of abnormal proliferation of vascular smooth muscle cells, and cardiovascular remodeling.^46^ However, the anti‐hypertensive action of the renal tissue KKS^47^ is not in the scope of current investigation.

Previous study^48^ has shown that nodose neurons functionally express B_1_R and B_2_R, especially the later one. Clinical evidence has demonstrated that the polymorphism of T‐58C and BE1 genotype of B_2_R are closely associated with lower BRS in either normotensive young subjects^49^ or hypertensive patients^50^ and BK‐dependent vasodilation.^51^ These observations imply that the modification of BK receptor expression is a crucial in BK‐mediated cardiovascular action. However, the expression pattern of BK receptors in the NG and NTS has not been evaluated so far under both physiological and hypertensive disease condition. The current immunoblotting results showed that a significant higher expression level for both B_1_R and B_2_R gene and protein was detected in either NG or NTS of control rats; and the cellular distribution of BK receptors was also confirmed by immunostaining analysis showing that the fluorescence of B_1_R and B_2_R were detected in myelinated (presumably A‐ and Ah‐types) and unmyelinated (presumably C‐types) afferent neurons. Intriguingly, a dramatic downregulation of both B_1_R and B_2_R in the NG and NTS were observed clearly in spontaneously hypertensive model rats with both genders, which explains that the decrease in BK‐induced BP reduction under hypertensive condition is due presumably to the downregulation of BK‐receptors. Even though immunostaining showed cellular distribution of B_1_R and B_2_R in myelinated neurons, the question remains to be answered if myelinated A‐ and Ah‐types act as a similar role in BK‐mediated BP reduction.

In this regard, whole‐cell patch experiment was conducted in identified BRNs isolated from adult female rats using our electrophysiological and pharmacological validations^6^, ^16^ and the membrane depolarization was tested in the presence of BK. Surprisingly, all tested myelinated A‐types were not respond to BK, whereas both myelinated Ah‐types and unmyelinated C‐types were significantly depolarized by BK, in which much lower concentration of BK is necessary for the depolarization in Ah‐types (30 nM) compared with C‐types (300 nM), the degree of depolarization is almost 2 folds in Ah‐types compared with C‐types, and the repetitive firings during initial phase of depolarization is only seen in Ah‐types, rather than C‐types. Consistently, BK‐induced neuroexcitation (membrane depolarization) was also confirmed by BK‐induced inward currents with strong degree and initial spikes due to Na^+^ channel activation in identified Ah‐type BRNs compared with C‐types.

## 5 CONCLUSION

Taken all together, the electrophysiological finding conjugated with functional and molecular observations, there datasets have demonstrated for the first time that female‐specific subpopulation of Ah‐type BRNs in the NG is a key player in BK‐mediated BP reduction via BK receptors, especially B_2_R, under both normotensive and hypertensive conditions. To explain why Ah‐type BRNs is more sensitive to BK, the future work of single‐cell qRT‐PCR using electrophysiologically identified Ah‐type neurons isolated from adult female rats would be needed to quantify the expression of BK receptors at the cellular level.

## CONFLICT OF INTEREST

The authors declare that there is no conflict of interest associated with the contents of this article.

## AUTHOR CONTRIBUTIONS

KXL, XXF, YF, RZ, and BYL designed the study and interpretation; KXL, XXF, AND YF conducted whole animal experiments; KXL, XS, YL, DW, LL, and CPC performed molecular experiments; KXL, XX, HDL, MZ, HLM, and YL analyzed the data; KXL, RZ, and BYL draft and revised the manuscript; KXL and BYL finalized the manuscript; YL, RZ, and BYL provided research funding.

## Supporting information

Supplementary Material

## Data Availability

The data that support the findings of this study are available from the corresponding author upon reasonable request.
