# Supplementary material for: Bradykinin‐mediated estrogen‐dependent depressor response by direct activation of female‐specific distribution of myelinated Ah‐type baroreceptor neurons in rats
Source: CNS Neurosci Ther. 2021 Dec 28;28(3):435–47. doi: 10.1111/cns.13792 (PMC8841294; doi:10.1111/cns.13792)
Supplement: Supplementary file 1 — Supplementary Material [file CNS-28-435-s001.doc]

**Online only Supplemental Materials**

**Bradykinin-Mediated Estrogen-Dependent Depressor Response by Direct Activation of Female-Specific Distribution of Myelinated Baroreceptor Neurons in Rats**

Ke-xin Li^1^, Yan Feng^1^, Xiong-xiong Fan^1^, Xun Sun^1^,Ying Li^1^, Di Wu^1^, Li Liu^1^, Chang-peng Cui^1^, Xue Xiong^1^, Hu-die Li^1^, Meng Zhou^1^, Hai-lan Ma^1^, Yang Liu^2^, Rong Zhang^1^, Bai-yan Li^1^

**Supplemental Tables**:

**Table S1: The list of all chemicals used in this experiment**

| **Name** | **Catalog #** | **Manufacture** |
| --- | --- | --- |
| L-NAME (N'-Nitro-L-Arginine-methyl ester) | WXBC9273V | Sigma-Aldrich |
| Pentobarbital Sodium | P11011 | Sigma-Aldrich |
| Heparin | 1170GR005 | BioFROXX |
| Sodium Nitroprusside | PHR1423 | sigma-Aldrich |
| Phenylephrine | P1240000 | Sigma-Aldrich |
| [ChloralHydrate](javascript:;) | B1826028 | Aladdin |
| Bradykinin | 3004 | TOCRIS |
| Sar-[D-Phe8]-des-Arg9-Bradykinin | 3230 | TOCRIS |
| [Phe8Ψ(CH-NH)-Arg9]-Bradykinin | 3229 | TOCRIS |
| TRIzol® | 15596-018 | Invitrogen |
| ReverTra Ace qPCR RT Kit | TYB-FSQ-101 | Toyobo |
| SYBR Green PCR Master Mix Kit | 24759100 | Roche |
| RIPA lysis buffer | P0013B | Beyotime |
| SDS | P0013G | Beyotime |
| Protease Inhibitor Cocktail | HY-K0010 | MedChemExpress |
| BCA Protein Assay Kit | P0010S | Beyotime |
| BSA | A-4503 | Sigma-Aldrich |
| Triton-X | 9002-93-1 | Sigma-Aldrich |
| Goat Serum | AR0009 | BOSTER |
| DAPI (4’,6-Diamidino-2-Phenylindole, Dihydrochloride) | C1005 | Beyotime |

**Table S2:** The list of all qRT-PCR primers used in this experiment

| **Gene List** | **Primers** | **Sequences** |
| --- | --- | --- |
| B1R | Sense primer | 5’-AGGCTAACGTGAGGTGAGGT-3’ |
|  | Antisense primer | 5’-CAGGGATTGGGTGGGAACTT-3’ |
| B2R | Sense primer | 5’-TGCTGTCGGGATGCTGGAAC-3’ |
|  | Antisense primer | 5’-TCACGTACACCAGCGGGTTGA-3’ |
| GAPDH | Sense primer | 5’-GAACATCATCCCTGCATCCA-3’ |
|  | Antisense primer | 5’-CCAGTGAGCTTCCCGTTCA-3’ |

**Table S3:** The list of all antibodies used in this experiment

| **Name** | **Catalog #** | **Manufacture** |
| --- | --- | --- |
| ANTI- B1R | ABR-011 | Alomone Labs |
| ANTI- B2R | ABR-012 | Alomone Labs |
| GAPDH | ac002 | Abclonal |
| IRDye® 800CW Goat anti-Mouse IgG (H + L) | 926-32210 | LI-COR |
| IRDye® 800CW Goat anti-Rabbit IgG (H + L) | 926-32211 | LI-COR |
| HCN1 | ab84816 | Abcam |
| Alexa Fluor® 488 goat anti-rabbit IgG (H + L) | A11034 | Life Technology |
| Alexa Fluor® 594 goat anti-mouse IgG (H + L) | A11032 | Life Technology |

**Table S4: The** **MAP reduction in the presence of different BK concentrations in adult male, age-matched female and ovariectomized (OVX) female** rats.

|  | **Concentration of BK** | | | | | |
| --- | --- | --- | --- | --- | --- | --- |
|  | 0.01 mg/ml | 0.05 mg/ml | 0.1 mg/ml | 0.25 mg/ml | 0.5 mg/ml | 1 mg/ml |
| **Male** | -6.5±1.14 | -6.9±1.11 | -8.4±1.6 | -10.9±1.22 | -13.03±1.47 | -16.58±1.35 |
| **Female** | -7.2±1.4** | -10.6±1.29** | -12.7±1.7** | -15.6±1.9** | -20.9±2.9** | -21.63±1.32** |
| **OVX** | -6.4±1.6^##^ | -6.3±1.4^##^ | -7.3±1.7^##^ | -9.7±1.6^##^ | -12.19±1.79^##^ | -15.98±0.69^##^ |

Date were presented as mean ± SD; ***P* < 0.01 *vs*. Male; ^##^*P <* 0.01 *vs*. Female.

**Table S5**: **The MAP reduction and recovery time in the presence of bradykinin receptor agonists in adult male and age-matched female.**

|  | **MAP reduction** | | **Recovery time** | |
| --- | --- | --- | --- | --- |
|  | **Male** | **Female** | **Male** | **Female** |
| **B_1_R agonist** | -8.67 ± 1.42 | -11.12 ± 2.12* | -13.71 ± 5.44 | -14.86 ± 6.15 |
| **B_2_R agonist** | -15.65 ± 2.19 | -20.73 ± 1.93** | -37.86 ± 11.16 | -60.00 ± 10.31** |

Date were presented as mean ± SD; **P* < 0.05 and ***P* < 0.01 *vs*. Male.

**Supplemental Figures**:

**Figure S1: Summary data of the MAP before and after BK microinjection in male (a), female (b), and ovariectomized (OVX, c) rats.** The averaged data were expressed as mean ± SD, **P <* 0.05 and ***P* < 0.01 *vs*. before, *n* = 7 rats.


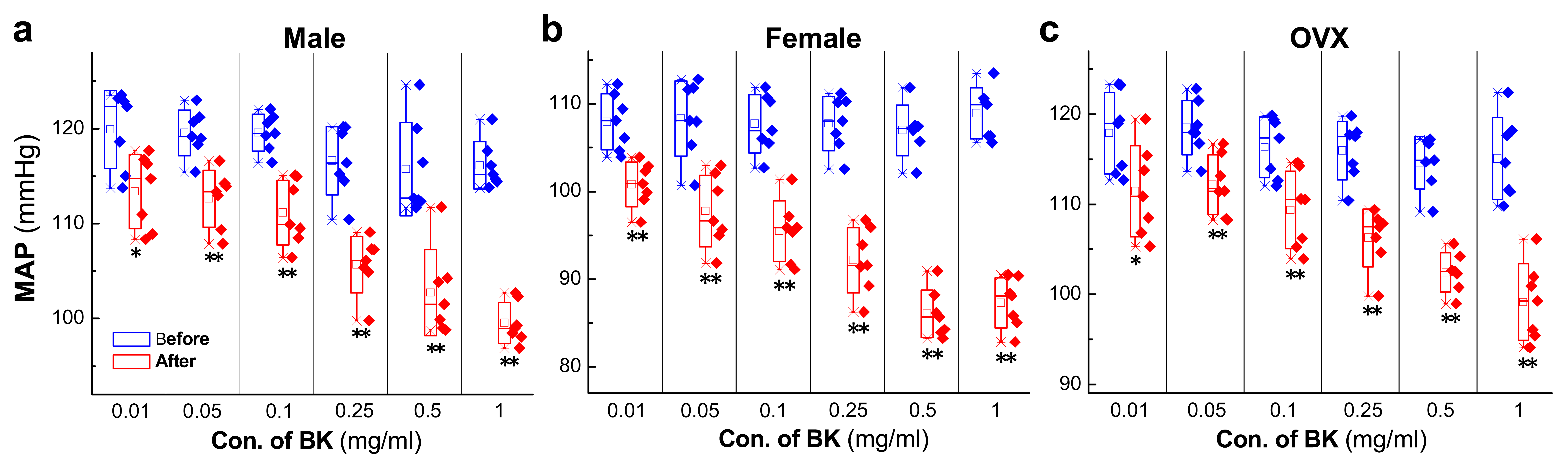


**Figure S2: Bradykinin receptors (BKRs) expression in NG and NTS in male, age-matched female and ovariectomized (OVX) rats.** (**A**) The mRNA expression of B_1_R in the NG and NTS tissues; (**B**) The mRNA expression of B_2_R in the NG and NTS tissues; Date were presented as mean ± SD, **P* < 0.05 *vs*. Male; ^#^*P <* 0.05 and ^##^*P <* 0.01 *vs*. Female, *n* = 4-5 from 4-5 rats.

**
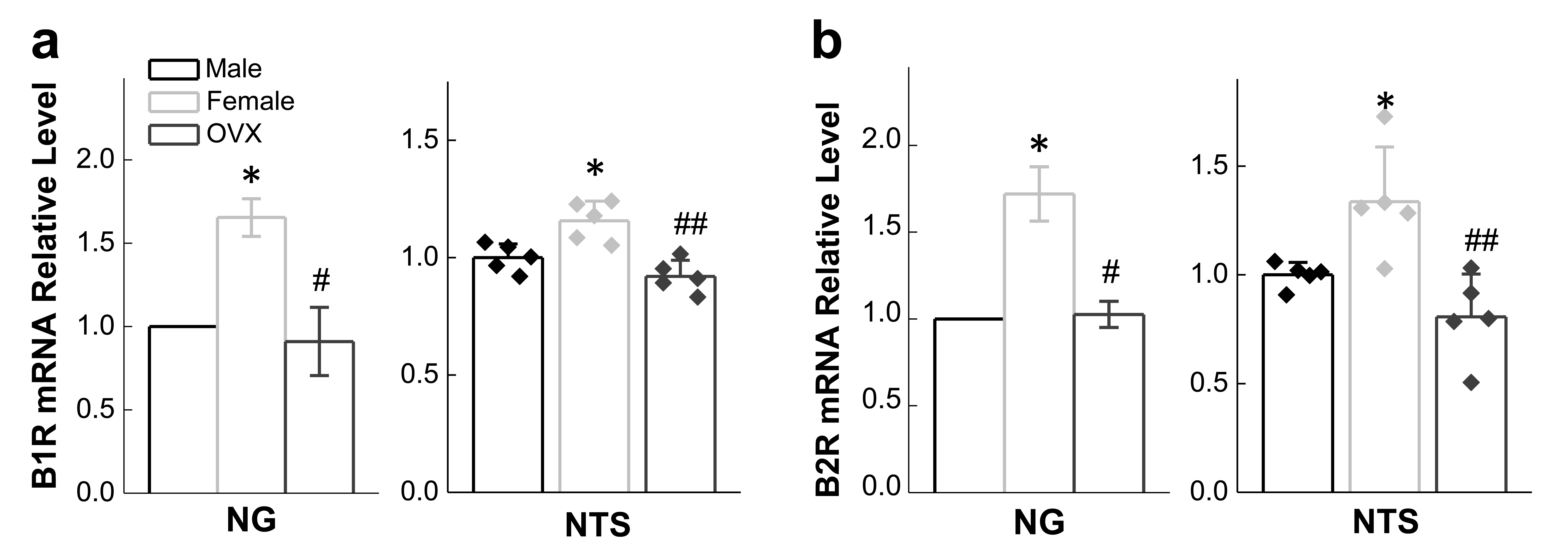
**

**Figure S3: Recording the systolic blood pressure (SBP) of hypertension model.** (**a**) L-NAME hypertension model was established by daily intraperitoneal injection of N'-Nitro-L-Arginine-methyl ester hydrochloride (L-NAME) (10 mg/200 g body weight) for four weeks. SBP was monitored during four consecutive weeks. Data were presented as mean ± SD; **P* < 0.05 and ***P* < 0.01 *vs.* F*-*Ctrl; ^##^*P* < 0.01 *vs.* M*-*Ctrl; ^&&^*P* < 0.01 *vs.* F*-*Ctrl*, n* = 8 rats. (**b**) Blood pressure recorded from spontaneously hypertension model of male and female rats (M-WKY, M-SHR, F-WKY, and F-SHR rats). Data were presented as mean ± SD; ***P* < 0.01 *vs.* WKY; ^##^*P* < 0.01 *vs.* M*-*WKY; ^&&^*P* < 0.01 *vs.* M*-*SHR*, n* = 6 rats.


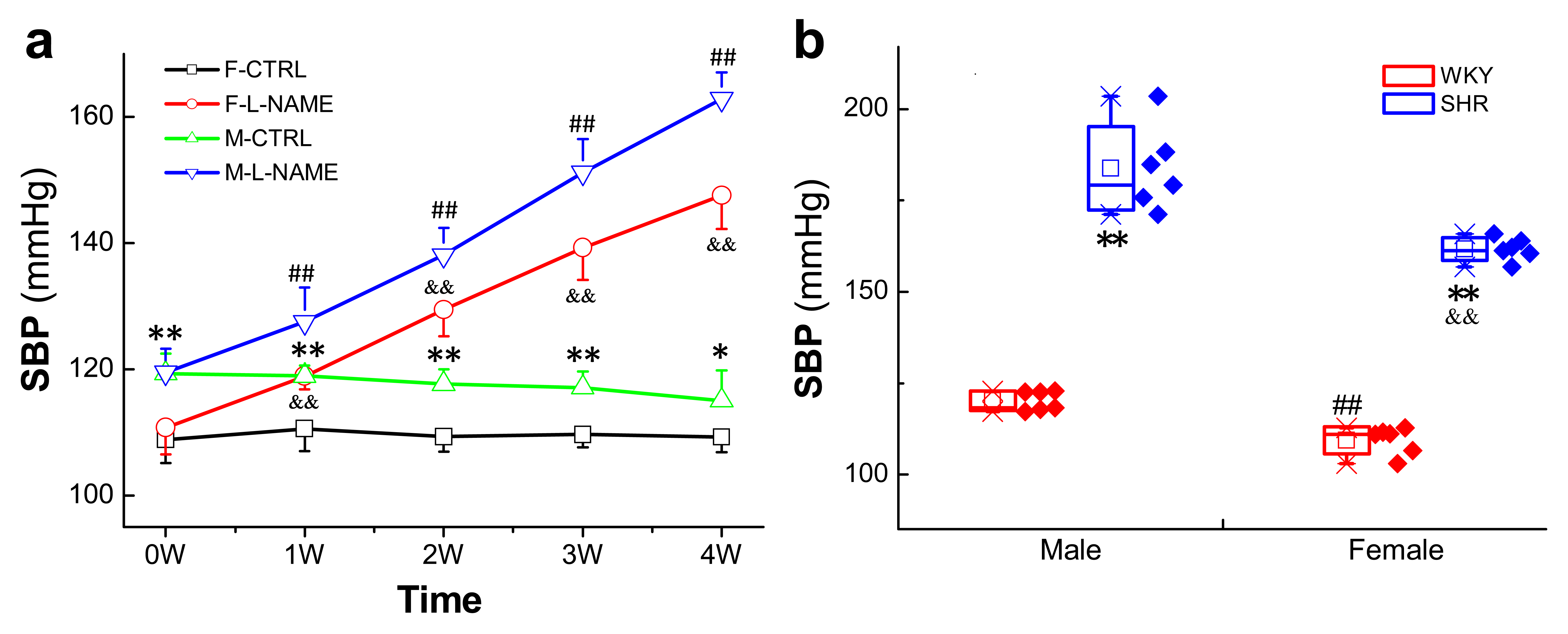


**Figure S4: The changes in baroreceptor sensitivity (BRS) of Secondary hypertension model.** The representative changes in MAP and heart rate (HR) were monitored in the presence of 1, 3, and 10 μg/kg sodium nitroprusside (SNP) or phenylephrine (PE). The summary changes of BRS (ΔHR/ΔMAP, bpm/mmHg) when treated with SNP or PE at different concentration in each group (the bottom panel). Data were presented as mean ± SD; **P* < 0.05 and ***P* < 0.01 *vs.* M-Ctrl*,* ^##^*P* < 0.01 *vs.* F-Ctrl, *n* = 7 rats for each group.


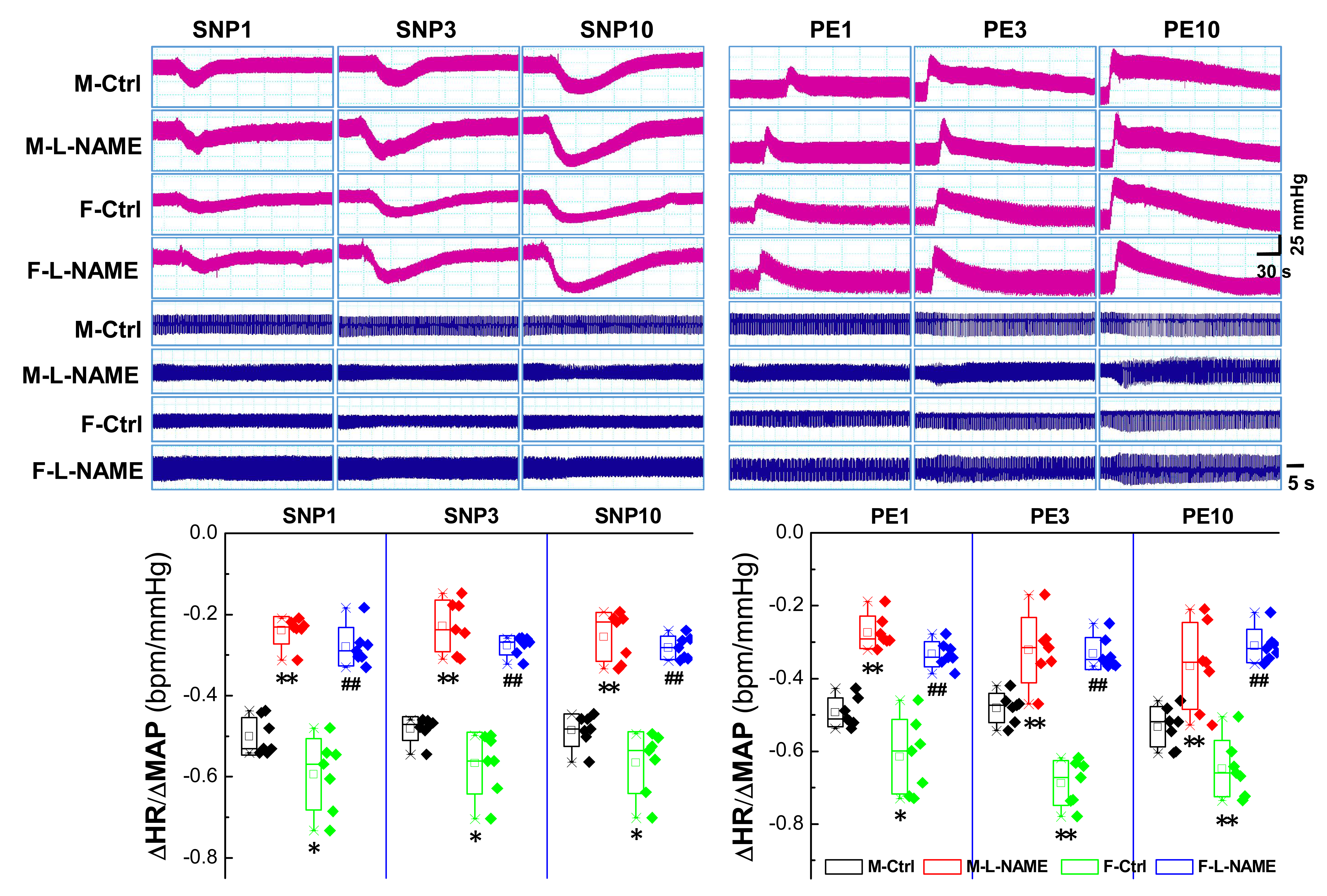


**Figure S5: Recording BRS in WKY and SHR model rats.** Averaged data were presented as mean ± SD, **P* < 0.05 and ***P* < 0.01 *vs*. M-WKY, ^##^*P* < 0.01 *vs*. F-WKY, *n* = 5


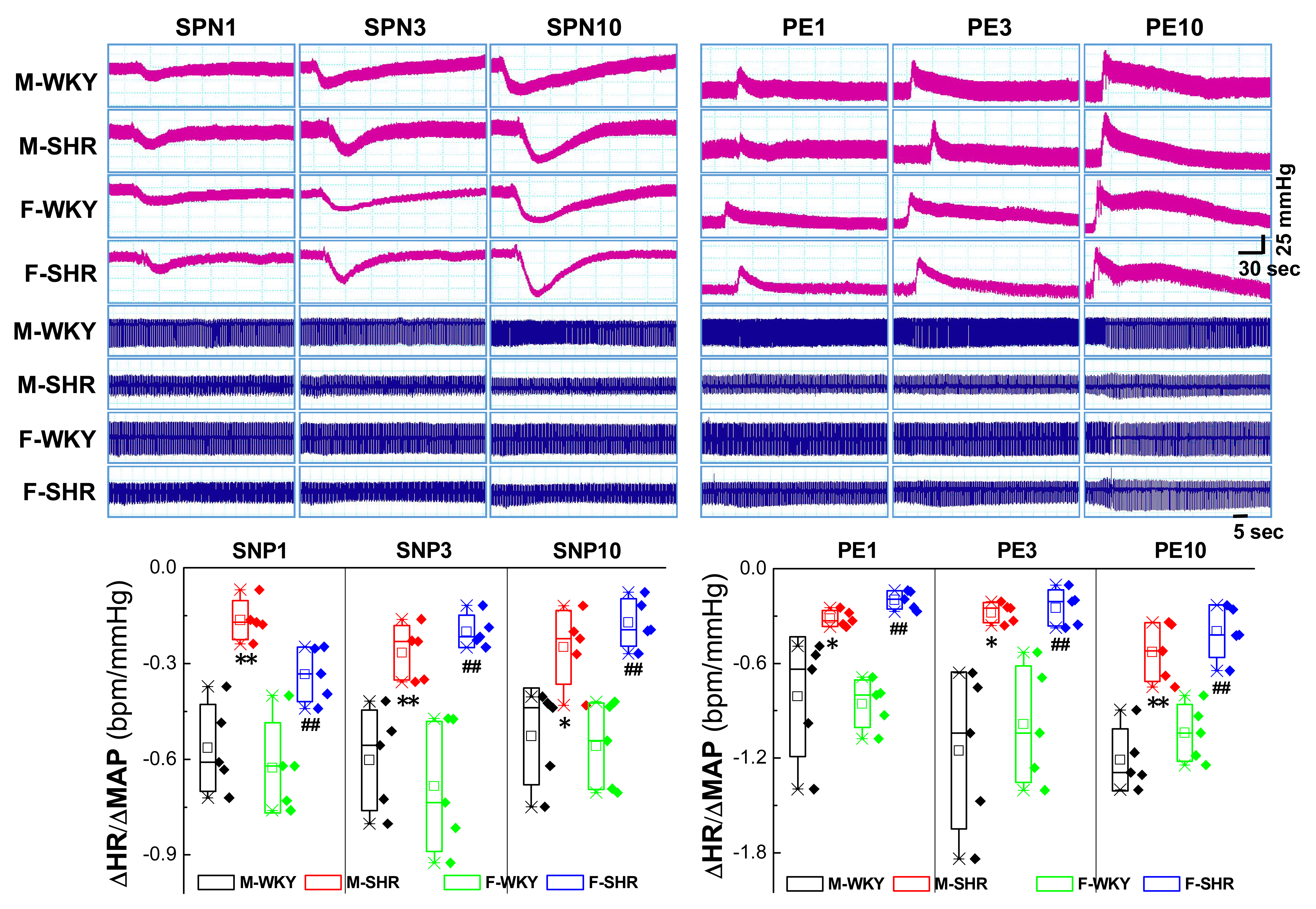


**Figure S6: Afferent fiber type of acute isolated nodose ganglia (NG) neurons validated by electrophysiological characteristics according to the conduction velocity (CV) generated from Vagus-nodose slice preparation.** The single action potential (AP) elicited by a brief pulse was recorded and the instant current changes over the course of membrane potential was also calculated and expressed as derivatives (dv/dt, mV/ms). (**a** & **d**): the representative AP and derivatives for myelinated A-types. For A-type, a brief AP duration less than 1.0 ms at 50% of height and negative hump presentation during repolarization could be used to distinguished to Ah- and C-type; (**b** & **e**): the representative AP and derivatives for myelinated Ah-types. For Ah-type, low AP firing threshold, fast up-stroke velocity, and narrow AP duration could be used to distinguished to C-type; (**c** & **f**): the representative AP and derivatives for unmyelinated C-types. The scale bars in C and F apply for all.

**
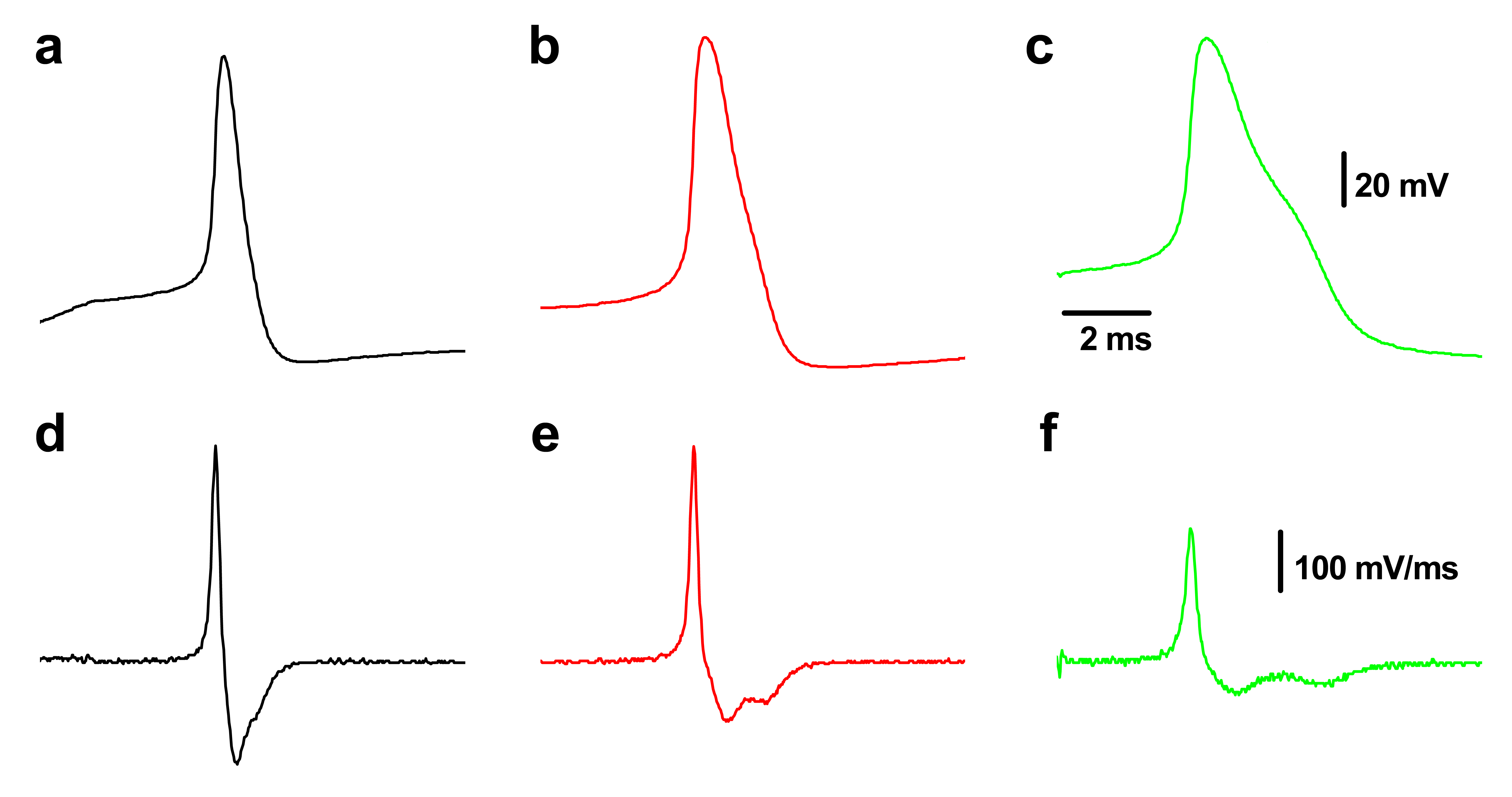
**

**Figure S7: Bradykinin (BK)-mediated inward currents in identified Ah-type neurons isolated from the nodose (NG) of adult female rats.** Single action potential (AP) was elicited under current-clamp mode for identification the afferent fiber type based upon the AP waveform characters. And then 30 nM BK (arrowhead)-induced inward currents were recorded (*n* = 16 recordings) using gap-free protocol under the voltage-clamp configuration. (**a** & **b**) two representative recordings with repetitive Na^+^ channel activations during the initial phase of inward currents.

**
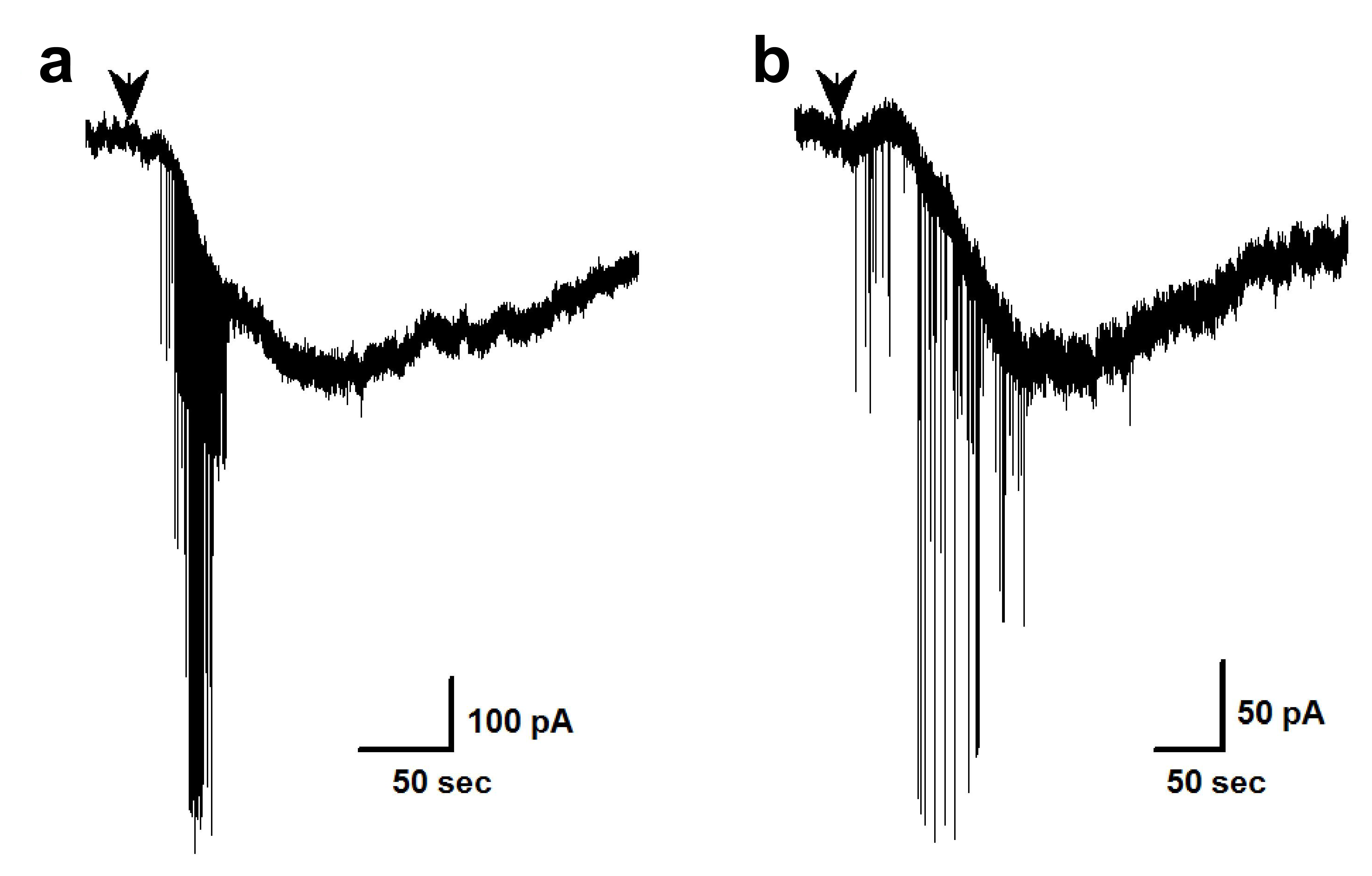
**

**Figure S8: Bradykinin (BK)-mediated inward currents in identified C-type neurons isolated from the nodose (NG) of adult female rats.** Single action potential (AP) was elicited under current-clamp mode for identification the afferent fiber type based upon the AP waveform characters. And then 300 nM BK (arrowhead)-induced inward currents were recorded (*n* = 16 recordings) using gap-free protocol under the voltage-clamp configuration. (**a** and **b)** two representative traces without repetitive Na^+^ channel activations during the initial phase of inward currents.

**
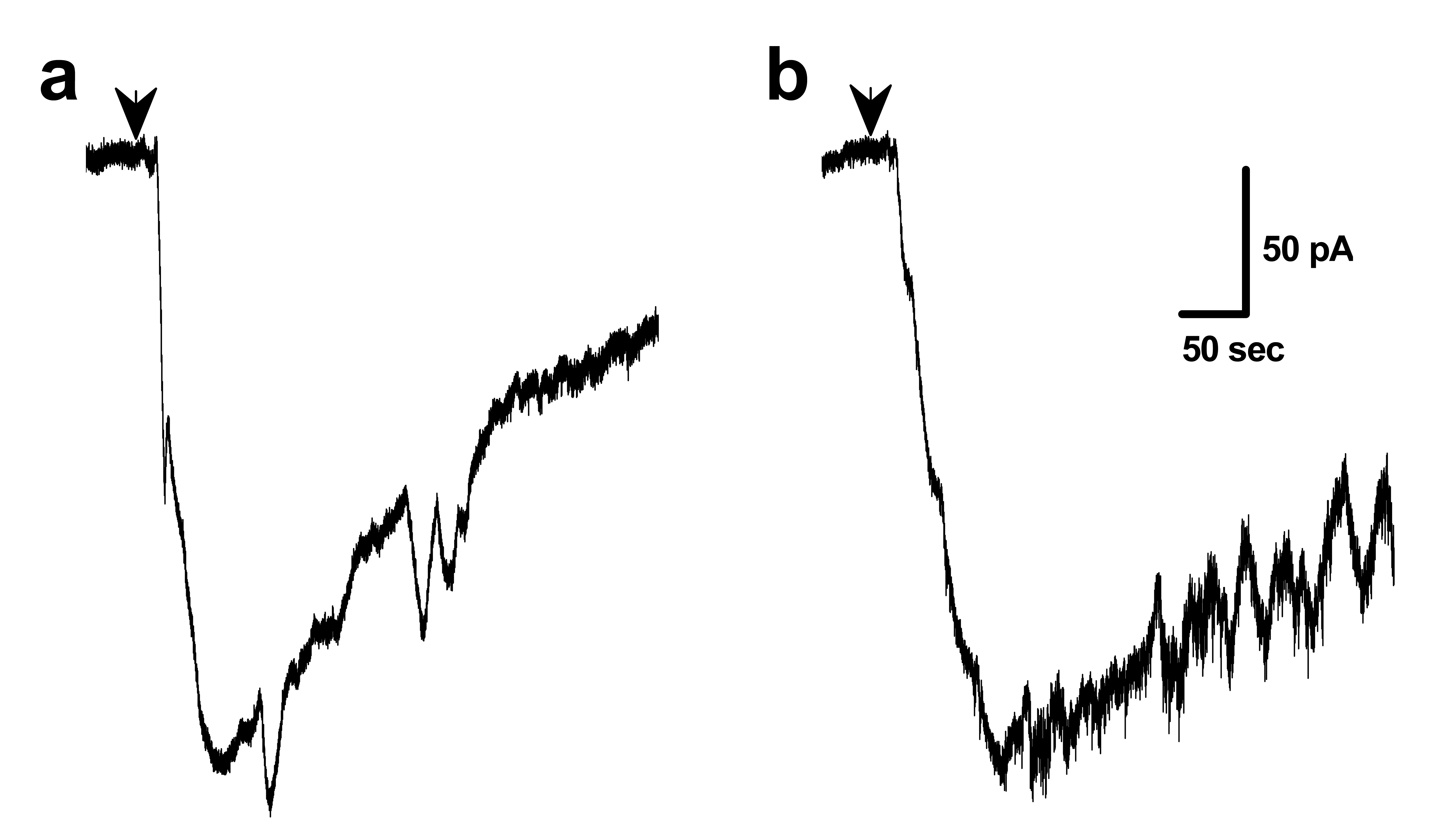
**
